# The Collaborative Payer Provider Model Enhances Primary Care, Producing Triple Aim Plus One Outcomes: A Cohort Study

**DOI:** 10.3390/healthcare5030048

**Published:** 2017-08-27

**Authors:** Thomas Doerr, Lisa Olsen, Deborah Zimmerman

**Affiliations:** Lumeris Inc., St. Louis, MO 63043, USA; olsen@lumeris.com (L.O.); dzimmerman@lumeris.com (D.Z.)

**Keywords:** medicare advantage, triple aim, health care value, health care costs, primary care, innovation

## Abstract

Rising health care costs are threatening the fiscal solvency of patients, employers, payers, and governments. The Collaborative Payer Provider Model (CPPM) addresses this challenge by reinventing the role of the payer into a full-service collaborative ally of the physician. From 2010 through 2014, a Medicare Advantage plan prospectively deployed the CPPM, averaging 30,561 members with costs that were 73.6% of fee-for-service (FFS) Medicare (*p* < 0.001). The health plan was not part of an integrated delivery system. After allocating $80 per member per month (PMPM) for primary care costs, the health plan had medical cost ratios averaging 75.1% before surplus distribution. Member benefits were the best in the market. The health plan was rated 4.5 Stars by the Centers for Medicare and Medicaid Services for years 1–4, and 5 Stars in study year 5 for quality, patient experience, access to care, and care process metrics. Primary care and specialist satisfaction were significantly better than national benchmarks. Savings resulted from shifts in spending from inpatient to outpatient settings, and from specialists to primary care physicians when appropriate. The CPPM is a scalable model that enables a win-win-win system for patients, providers, and payers.

## 1. Introduction

Traditional payers and doctors have sometimes clashed, with tactics including payment delays [1], deception [2], micromanagement [3], rationing care through inconvenience [4], brinksmanship [5], and litigation [6]. The Triple Aim requires that doctors take on attributes of payers (fiscal responsibility), and payers act more like providers (with an emphasis on the patient’s experience of care). In recent years, some providers and payers are beginning to chip away at years of built-up distrust through new partnerships [7]. However, there are few studies with detailed data available on outcomes.

Aetna and NovaHealth, an independent physician association based in Portland, Maine, developed a payer-provider collaborative care model for a case study of 750 mutual Medicare Advantage members [8]. Their model has three core areas: dedicated care management resources, data reporting, and quality management. The Ambulatory Intensive Caring Unit model typically requires payer financial collaboration to provide risk-adjusted flat fee reimbursement for primary care [9]. The Extensivist model requires more substantial restructuring of traditional fee-for-service reimbursement, as providers are typically paid salaries plus performance-based bonuses [10,11].

In their seminal work on the Triple Aim, Berwick et al. defined the crucial role of the integrator [12]. An integrator is an entity that accepts responsibility for all three components of the Triple Aim for a specified population. The authors speculate that some entities, without unified financing or a single delivery system, could take on a strong integrator role, and that such a role might be within the reach of a “powerful, visionary insurer”. Independent of Berwick’s work, we developed a model in which the payer functions as a full-service collaborative ally of multiple physician organizations, while each maintains their independence. This study provides a detailed exposition of the methods, processes, and outcomes of this collaboration.

In 2004, a physician-founded and -owned Medicare Advantage plan in St. Louis enrolled its first members. In 2007, this health plan accepted outside investors, and by 2014 the physician ownership share had decreased below 15%. In 2008, the management of the health plan published the Collaborative Payer Model (CPM) white paper [13]. The CPM was a blueprint for organizations led by providers, health systems, or traditional payers to succeed in value-based care. As the model became further refined, the providers’ responsibility to collaborate was incorporated into the model’s name and acronym, which became the Collaborative Payer Provider Model and CPPM, respectively. The “provider” in the CPPM refers to primary care professional-led teams. The professionals could be physicians, physician assistants, nurse practitioners, or registered nurses.

## 2. Study Methods Part One: The Collaborative Payer Model

Table 1 lists the three main elements of the CPPM and eight critical success factors. Physician engagement and the Accountable Primary Care model [14] make up the final common pathway through which these success factors flow. While the Medicare Advantage plan described here is one of successful instantiation of the CPPM, it also applies to other value-based populations such as Medicaid, health insurance exchanges, and Accountable Care Organizations (ACOs).

### 2.1. Radical Alignment of Incentives between Payer, PCPs and Patients

Aligned incentives are designed to reduce conflicts of interest in value-based care. In a typical CPPM contract in Medicare Advantage, the payer might allocate 15% of total revenue to cover the costs of their payer functions. Claims are paid from the remaining 85% of the revenue. If claims costs are less than 85% of total revenue, the residual is shared with primary care provider (PCP) organizations. Accountable primary care can be delivered by primary care physicians, provider-led teams, or by medical organizations. In a typical full risk contract, the PCP organizations might receive 60% of the share back, with opportunity to earn up to 80% of the share back if quality and other contractual incentives are met. The payer receives the remaining 20–40% of the share back. Aligned patient incentives make appropriate care affordable, and supply-sensitive care relatively expensive.

### 2.2. Transparency

The payer provides complete financial, clinical, and provider comparative performance data transparency in a timely manner. The flow of revenue and risk adjustment from the Centers for Medicare and Medicaid Services (CMS) through the payer to paid claims, and the distribution of residual funds and quality bonuses is available for PCP scrutiny. This forms the foundation for trust.

The payer finances and supports a data platform that provides updates on a nightly basis, including performance against contract incentives. The PCPs provide data from their electronic health records to augment the payer’s data from across the continuum of care. The payer runs comprehensive analytics and provides actionable patient-specific rules-based messages for PCP-led teams that improve the quality and efficiency of care. Comparative performance at multiple population levels allows for benchmarking, sharing of best practices, and healthy competition.

### 2.3. Reciprocal Responsibilities and Accountability

PCPs are held accountable through contract incentives, payer data-mining for outliers, random and targeted chart audits, and through member appeals, grievances, and disenrollment interviews. A complete listing of the PCP’s responsibilities has been codified into the Accountable Primary Care model’s 9Cs.

The payer’s responsibilities are to provide technology, data, decision support, services, education, and consulting, which all lead to the virtual integration of care delivery. Payers are accountable to the CMS through a multitude of audits, reports and Star ratings. More than half of the Star ratings are quality of care measures. Table 2 summarizes the major differences between traditional payers and the CPPM.

While the Medicare Advantage plan described here is one successful instantiation of the CPPM, it also applies to other value-based populations such as Medicaid, health insurance exchanges, and ACOs. While HMO is the preferred type of insurance product, high-value care can also be delivered within Preferred Provider Organization (PPO) populations. The PPOs must implement consumer-driven incentives that direct members to utilize high-value providers, and they must strongly incentivize members to make in-network PCP selections and engage those PCPs in the Accountable Primary Care model.

## 3. Study Methods Part Two: Data

After filtering, Essence Healthcare, a Medicare Advantage plus Part D health plan (#H2610) averaged 30,561 members over the five-year period (Table 3). The plan is not part of an integrated delivery system; instead, it relies on a PCP gatekeeper model with narrow networks of independent providers. The providers of care were similar from 2010–2014. The 1200 specialists were paid above fee-for-service (FFS) Medicare rates to compensate for their high-value care and collaboration with PCPs. The plan contracted with 22 hospitals, of which seven were independent, and 15 were in three hospital systems. Approximately 350 PCPs contracted with the plan. 

Two cohorts were defined for comparison. The Essence, or CPPM cohort, consisted of all members of the health plan who were 65 years and older. The 5% FFS cohort consisted of a sample of Medicare FFS data matched for age, gender, and county. The Aon corporation was hired to independently define the cohorts, and to categorize, analyze, and filter the data to assure objectivity. Aon filtered (removed) the following types of members from both cohorts because their frequencies were substantially different: end stage renal dialysis, dual eligible, disabled members, FFS members not enrolled in both Medicare Parts A and B during the calendar year, and members with costs exceeding $250,000 in one year. 1,579 members from the health plan were removed. Medicare Part D drug data were excluded from the analysis. Expenditures on specialists were left at their actual payments (105% of FFS Medicare). Claims data for the two filtered populations were studied for each of the five years. Each type of filtering and the risk adjustment factor normalization reduced the apparent CPPM savings. Using two-tailed Student’s *t*-tests, we tested for differences in per member per month costs for each of the five study years.

## 4. Results

During calendar years 2010–2014, the CPPM health plan in Missouri and southern Illinois (Essence Healthcare) had costs that averaged 73.4% of a matched FFS Medicare cohort adjusted for age, gender, and county. Table 3 contrasts this CPPM health plan with the matched 5% FFS population for calendar years 2010–2014.

Table 4 compares the matched cost data for members and beneficiaries continuously enrolled for the entire 60-month period. The costs of the continuously enrolled Essence cohort were lower than all filtered members relative to the FFS Medicare continuously-enrolled beneficiaries cohort.

**Figure 1:**
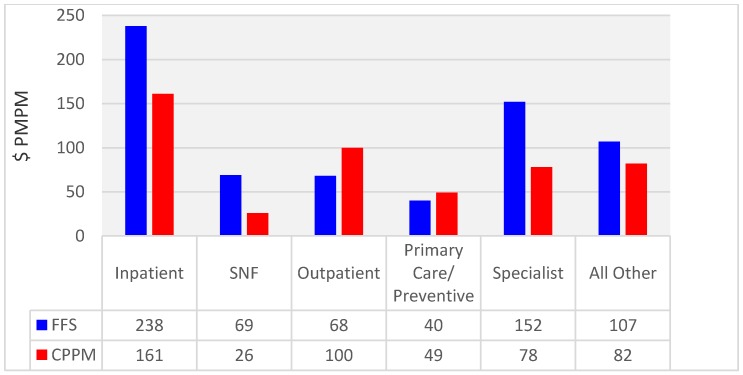
2010 through 2014 Costs by Service Category ($PMPM). CPPM is the Collaborative Payer Provider Model. FFS is the 5% Limited Data Set from Fee-For-Service Medicare. PMPM is Per Member Per Month costs. SNF is Skilled Nursing Facility. PMPM costs were risk-adjusted to a 1.00 risk basis to make them comparable between FFS and CPPM.

Figure 1 is an analysis based on classifying costs into one of six groups. In Figure 1, the cost of accountable PCP visits and preventive services was 121% of the FFS unmanaged control group. This underestimates the increase in primary care services, because it does not account for the many CPPM services (for example, virtual visits, care coordination) that were not reimbursable in the FFS remuneration system. This was offset by the 48% decrease in the total specialist costs. Inpatient bed days were 32% lower and skilled nursing facility days were 62% lower.

This health plan was rated at 4.5 Stars [15] by CMS in 2012–2015, and 5 Stars in 2016 based partially on 2011 through 2015 Health Care Effectiveness Data and Information Set (HEDIS)/Star quality metrics for patient care delivered in 2010 through 2014 along with patient experience, access to care, and care process metrics. Nationally, about 13% of plans are typically rated 4.5 Stars, and 3% rated 5 Stars each year. The CPPM health plan was the only 5-Star Medicare Advantage plan in Missouri and Illinois. Of particular note, for the Consumer Assessment of Health Plans’ member survey questions C24 (getting needed care) and C28 (health plan quality), the plan averaged 4.95 stars over the five study years. The plan was also rated 5 Stars each year for C27 (low percentage of members choosing to leave the plan).

In 2011, DSS Research surveyed the PCPs contracted with this MA plan. Of the 102 PCP respondents (response rate 44%), 90.2% would probably or definitely recommend this MA health plan to another physician. This is significantly higher than the national average (83.8%, *n* = 4529) at the 95% confidence level [16,17]. In 2010, DSS Research surveyed specialists contracted with this MA plan. Of the 169 specialist respondents (response rate of 19.4%), 91.4% were satisfied or very satisfied with this plan. This is significantly higher than the national average (88.9%, *n* = 5810) at the 95% confidence level.

If the PCPs were reimbursed with $80 per member per month (PMPM)—twice the typical FFS Medicare remuneration—the MA plan would have medical cost ratios of 73.4% in 2010, 72.2% in 2011, 72.5% in 2012, 77.9% in 2013, and 79.4% in 2014 before surplus distribution. These low medical cost ratios occurred despite having the most generous member benefits of any zero-dollar premium MA health maintenance organization in its market. The maximum out-of-pocket expenses were $2025, while the next lowest were $2575, calculating plans from 2010–2014. Goroll et al. modeled the cost of comprehensive primary care for patients with medium need/risk at $67 PMPM [18]. The CPPM is able to attain these financial outcomes even if PCPs are paid $80 PMPM—twice typical FFS Medicare remuneration.

## 5. Discussion

As Figure 1 illustrates, with the CPPM, care is shifted from inpatient to outpatient and from specialists to PCPs. In Table 3 and Table 4, the increases in costs in 2013 and 2014 were exacerbated by sequestration, the reduction in Medicare Advantage to parity with FFS Medicare, and changes in the CMS-HCC coding model. The utilization as evidenced by the bed days per thousand members is an indicator that physician performance was relatively stable during the last two years of declining revenue and increasing unit costs.

Fischer and Wennberg have estimated that over 50% of all Medicare spending in the last two years of life is “supply-sensitive,” and half of it has no demonstrable value [19]. The CPPM and the Accountable Primary Care models, driven by PCPs who are appropriately incentivized, armed with information transparency and decision support tools, provide practical methods for better distinguishing supply-sensitive care from necessary care, and directing patients to high-value care. Utilizing a different methodology, the Institute of Medicine has estimated that 30% of health care spending is waste [20]. Independently, Donald Berwick affirmed estimates in this range. He noted that much is done in health care that does not benefit patients at all, and many physicians know it [21]. Meanwhile, in a 2015 survey, the most common fear of seniors about retirement was high medical costs. It was ranked #1 by 28% of Americans [22]. The MA plan’s relatively low maximum out-of-pocket expense limit protects patients from the coinsurance, which can be bankrupting in catastrophic illnesses without costly supplemental FFS Medicare insurance.

The average 4.95-Star ratings in the Consumer Assessment of Health Plans’ member survey for questions number C24 (getting needed care) and C28 (health plan quality), and 5-Star rating in C27 (low percentage of members choosing to leave the plan) attest to superior patient satisfaction despite the removal of unnecessary spending.

Metrics of physician satisfaction were significantly above national benchmarks. Several thought leaders such as Bodenheimer [23] have articulated physician satisfaction as the fourth aim—the Triple Aim *Plus One*.

The outcomes from other models of care are less compelling than the CPPMs. While Aetna and NovaHealth’s reported intermediate outcomes metrics were encouraging, the relevant zero-dollar premium MA plan, Aetna Medicare Value Plan (#H3597-001-0), had a maximum out-of-pocket expense of $6700 in 2015. This contrasts with average maximum out-of-pocket expenses of $2025 for the CPPM. Aetna’s plan was rated 4 Stars in 2012, 2014, and 2015, and 4.5 Stars in 2013 [24]. In an incomplete implementation of the Ambulatory Intensive Caring Unit, Boeing realized savings of 20% after accounting for the costs of care delivery [25]. Both CareMore and Alignment Healthcare’s [26] Extensivist models have reduced many of the different rates of utilization relative to CMS averages, but the overall savings amounts are not available for either model.

Many reforms expect PCPs to bear the costs, while the benefits accrue elsewhere. For example, Deloitte estimated that the costs of the patient-centered medical home to be $148,347 to $163,347 annually per primary care physician [27]. In our implementations of the CPPM, we find that the $80 PMPM allocation for primary care costs is necessary to cover their costs, and to enable them to be able to afford to get off their hamster wheels [28]. Only then can they create far more than $80 PMPM in value by removing wasteful supply-sensitive Medicare spending. The $49 PMPM in Figure 1 only captures the PCP charges and preventive care that are billable under traditional FFS Medicare. The other $31 PMPM comes from part of the share back.

Notably, these CPPM outcomes were not obtained within an integrated delivery system. The contracted physicians practiced in a physician-hospital organization, several independent physician associations, and medical groups of varying size and cohesiveness. In fact, the CPPM’s virtual integration is designed for providers who are not part of an integrated delivery system.

In scaling up this model, we encountered resistance from many payers who found the radical transparency and alignment of incentives of the model to be too much of a shock to their traditional relationships with providers. Nonetheless, we typically launch about four new Medicare Advantage plans per year. We start two per year in a long-term partnership with a national insurance company. And we partner with a couple of provider groups or hospital systems per year (many are already ACOs) that want to stand up new Medicare Advantage plans. From these foundations, we are extending our efforts into other products, such as Medicare PPO, Medicaid, and commercial insurance. Barriers to scaling up the model include: resistance to doubling the funding of primary care, the tentative industry transformation from volume to value-based reimbursement, and the limited (though increasing) acceptance of global capitation.

Despite the declining numbers of independent physicians, the shift from volume- to value-based reimbursement makes the CPPM more relevant today than when it was articulated in 2008. In our experience, a variety of compensation models and share back levels generate Triple Aim Plus One outcomes as long as the remainder of the CPPM and its critical success factors are implemented. Meanwhile, global capitation is spreading to Medicaid and Health Insurance Exchanges. While the upcoming changes to FFS Medicare reimbursement are directionally correct, the planned compensation and bonuses neither cover the costs of comprehensive primary care, nor adequately incentivize the extra work needed to create high-value care. The CPPM does both, while offering the country lower costs of care.

This study has the following limitations. First, it utilized referrals and narrow specialist networks in a Medicare Advantage plan. Of course, the members were well aware of the need for referrals before joining the plan, but the applicability of the CPPM to preferred provider organization models of insurance is unknown. While the study utilized global risk contracts, these are a core feature of the radical alignment of incentives between provider, payer, and patient. Furthermore, the excellent Star ratings are a good proxy for quality, but we have no direct comparison of quality measures between the two cohorts.

Since there is no randomized control group, only a matched FFS Medicare cohort, it is possible that it is a post hoc fallacy to assert a cause and effect relationship between the CPPM and the five years of outcomes data in this study. Yet the relationship between the tenets of the CPPM and the outcomes is plausible. The radical alignment of incentives is a powerful motivator for PCPs and patients to remove waste from health care. Unprecedented transparency provides the foundation for trust, as well as collaboration on responsibilities and accountability. Such collaboration recaptures value destroyed by entrenched ideologically adversarial positions.

In addition to the primary outcomes of the CPPM enumerated in the abstract, there are a number of other advantages. The CPPM offers: (1) enhanced access to primary care and more time with PCP-led teams; (2) new hope for the viability of primary care, which is vital for the creation of high-value care; (3) PCP stewardship of the patient’s health and health resources as distinguished from a traditional HMO gatekeeper role; and (4) recapture of the value destroyed in adversarial payer–physician relationships. With proper compensation in global risk contracts, PCPs are liberated from the traditional constraints on the time, place, and manner of care. They have the flexibility to meet their patients’ needs in the most rational, cost-effective ways available.

## 6. Conclusions

The CPPM reduces unnecessary supply-sensitive care, which enable savings that support the richest member benefits in the market. Narrow PCP networks with engaged physicians managing the total costs of care make necessary beneficial care more affordable. In addition, physicians are compensated at above-market rates, and the overall medical cost ratios average 75.1%. Patient’s perception of quality and satisfaction with care, as measured through Star ratings, are superior. Physician satisfaction is above industry benchmarks. Thus the CPPM approaches the Triple Aim *Plus* One. It is a promising template for risk-adjusted capitation and other value-based contracts outside of integrated delivery networks.

## Figures and Tables

**Table 1:** The Collaborative Payer Provider Model (CPPM)’s three main elements and eight critical success factors.

CPPM Element	Critical Success Factor
**I**	**Radical alignment of incentives between payer, physicians, and patients**
	(1) Total cost of care incentive for Primary Care Providers (PCPs). (2) Substantial quality incentive for PCPs. (3) Internal physician compensation formulas produce palpable rewards for delivering high-value care. (4) Primary Care Physician aggregation into groups in order to produce an actuarially credible number of lives (1500–2000). (5) Narrow PCP network producing PCP panel density, which intensifies incentives and enhances PCP engagement.
**II**	**Complete clinical, financial, and comparative performance transparency**
	(6) PCP visibility into cost, quality, comparative performance, and care across the continuum.
**III**	**Reciprocal responsibilities and accountability**
	(7) The delivery system must deliver Accountable Primary Care. The payer is accountable to the Centers for Medicare and Medicaid Services (CMS), members, and providers. (8) Medical group leadership facilitates cultural transformation and implementation of the CPPM.

**Table 2:** Differences between traditional payers and collaborative payers.

Item	Traditional Payer	Collaborative Payer
**(1) Alignment of Incentives**		
Risk sharing	In the late 1990s, the payers typically gave all the risk to the doctors and had little interest in their success. The payers cared about the payers’ outcomes, such as membership and revenue growth.	Radical alignment of incentives. Providers get up to 80% of the share back, and payers 20% of the share back. The payer is only financially successful if the provider is successful. The collaborative payer cares about PCP outcomes because their economic fates are linked together.
Incentives	Providers are rewarded for increasing volume of care.	Providers (and the payer) are rewarded for increasing the value of care. Up to 25% of share back may be based on quality and patient satisfaction metrics.
Quality Incentives	Typically too many metrics with inadequate funding. For example, a 2% bonus for 50 measures.	10 to 15 achievable metrics. Up to 20% of the share back for improvement and for absolute performance.
Contracting	Adversarial contracting and unit cost management. Zero sum negotiations.	Collaborative contracting. Win-win negotiations, as the 30% of health care spending that is waste is decreased.
**(2) Transparency**		
Clinical and financial data sharing	Minimal information sharing. When shared, typically too late for interventions. Information asymmetry is exploited for the payer’s advantage.	Complete clinical, financial, and comparative performance data transparency as soon as available.
**(3) Responsibilities and Accountability**		
Customer	Members and employers	CMS/employers, members, physicians
Provider network strategy Provider network strategy (con’t)	Large networks to increase sales and revenue, contract with everybody, hammer down unit costs. Pass cost increases onto employers.	Narrow or preferred networks, PCPs recommend particular specialists for inclusion in the networks. Limit the number of PCPs to increase their engagement with the payer.
Cost management	Traditional burdensome utilization management. Cost increases are passed onto the employers.	Referrals usually only for notification and communication. Utilization management is focused and coordinated. Wasteful care is reduced, creating profitability.
Payer relationship with physicians and members	Payers go around the doctors to care for their members.	Mutual dependence; collaborative payer supports the doctor–patient relationship. All three work to reduce waste and increase quality.
Payer contracts with vendors who provide care for patients	Has many vendor contracts to meet their members’ needs. They go around the PCPs and directly provide care to members.	Minimizes these. Collaborates with PCPs to mutually approve a few vendors. Honors the PCP–patient (member) relationship.

**Table 3:** Filtered CPPM membership and costs compared to Matched Fee-For-Service Medicare (FFS).

Year	# CPPM Members	CPPM Costs ^†^	CPPM MCR *	CPPM BD/K	CPPM RAF	#FFS Patients	FFS Costs ^†^	FFS RAF	CPPM/FFS Costs ^†^	*p* Value
2010	24,054	$485	73.4%	1188	1.09	8827	$672	1.18	72.2%	<0.001
2011	27,898	$482	72.2%	1181	1.11	8878	$675	1.16	71.4%	<0.001
2012	31,143	$476	72.5%	1138	1.14	8804	$682	1.17	69.8%	<0.001
2013	33,195	$498	77.9%	1113	1.13	8815	$671	1.12	74.2%	<0.001
2014	36,516	$540 ^‡^	79.4%	1151	1.05	8350	$672	1.12	80.4%	<0.001
**Average**	**30,561**	**$496**	**75.1%**	**1154**	**1.10**	**8375**	**$674**	**1.15**	**73.6%**	

CPPM is the Collaborative Payer Provider Model. FFS is the 5% Limited Data Set from Fee-For-Service Medicare. MCR is Medical Cost Ratio. RAF is the CMS-HCC Risk Adjustment Factor. BD/K is Bed days per thousand members per year. * The MCR is computed by the authors based on assumed $80 per member per month primary care physician costs in the CPPM. ^†^ Costs are per member per month, which were risk-adjusted to a 1.00 risk basis to make them comparable between FFS and CPPM. ^‡^ In 2014, CPPM costs of $540 are due to a major change in the CMS-HCC risk model. The *p* values are from Student’s t tests comparing the CPPM and FFS costs each year.

**Table 4:** Continuously Enrolled CPPM Members for All Five Years’ Per Member Per Month (PMPM) Costs Compared to Matched FFS Beneficiaries *.

Year	CPPM Cohort ^†^	FFS Cohort ^†^	CPPM/FFS Cohort ^†^
2010	$370	$561	66.0%
2011	$360	$559	64.4%
2012	$351	$562	62.5%
2013	$373	$576	64.8%
2014	$442	$620	71.3%
**Average**	**$379**	**$576**	**65.8%**

CPPM is The Collaborative Payer Model payer. FFS is the 5% Limited Data Set from Fee-For-Service Medicare. PMPM is Per Member Per Month costs. * PMPM costs were risk-adjusted to a 1.00 risk basis to make them comparable between FFS and CPPM. ^†^ These cohorts were comprised of patients who were members of the health plan for all five years to matched FFS Medicare beneficiaries continuously enrolled for all five years.
